# Cultural Adaptation of the Modified Mini‐Mental State Test and Rowland Universal Dementia Assessment Scale in Shawi Native Communities of the Peruvian Amazon

**DOI:** 10.1002/alz70860_104051

**Published:** 2025-12-23

**Authors:** Alicia Boluarte Carbajal, Arantxa Sánchez Boluarte, Danilo A Sanchez Coronel, Monica M Diaz

**Affiliations:** ^1^ Universidad Cesar Vallejo, Los Olivos, Lima, Peru; ^2^ Universidad Peruana Cayetano Heredia, Lima, Lima, Peru; ^3^ School of Public Health. University of Washington, Seattle, US, WA, USA; ^4^ Instituto Nacional de Ciencias Neurologicas, Lima, Lima, Peru; ^5^ University of North Carolina at Chapel Hill School of Medicine, Chapel Hill, NC, USA

## Abstract

**Background:**

The prevalence of dementia is estimated to be 1–4% among native Amazon communities. Our study aimed to culturally adapt the Modified Mini‐Mental State Exam (3MS) and the Rowland Universal Dementia Assessment Scale (RUDAS) for the Shawi Amazonian community in Peru, a Shawi‐monolingual community of low socioeconomic status with very high illiteracy rates.

**Method:**

We followed the International Test Commission guidelines for cultural adaptation, combining focus groups discussions (FGD) of community members, stakeholder cognitive interviews and expert judgement. Ten interviews were conducted with healers, community leaders (“apus”), older adults, and Spanish‐Shawi bilingual teachers to gather cultural insights. FGDs facilitated understanding of Shawi traditions and daily life, which informed the adaptation of each test item and administration protocols. Eight clinical experts, including general practitioners, neurologists and psychologists, externally validated the content. Native bilingual translators performed forward and backward translations, ensuring semantic equivalence and eliminating cultural bias. A pilot study with 20 participants was conducted to refine instructions, resolve ambiguities and standardize administration.

**Result:**

FDGs and interviews highlighted key cultural elements, such as the use of lunar phases for temporal orientation and the importance of communal labor (“faena”) and reliance on the “apu” to assess judgement. Test items related to language, attention and similarities were adjusted to reflect activities of daily living, including items commonly encountered in day‐to‐day life. The Shawi historically did not have a written language, we used alternative questions for those who were illiterate, adapted from a study in native Amazonians from Bolivia. The translation process incorporated culturally relevant elements, ensuring the adapted instruments aligned with the Shawi context. The pilot study resolved comprehension issues, determined administration times and informed the creation of an application manual for field use.

**Conclusion:**

This 3MS and RUDAS adaptation represents an important step in enabling accurate cognitive assessments among monolingual indigenous communities with high illiteracy rates. The resulting tools provide culturally tailored measures essential for early detection of cognitive impairment and targeted interventions in indigenous communities.

